# Sex-Specific Relationships Between Interoceptive Accuracy and Emotion Regulation

**DOI:** 10.3389/fnbeh.2020.00067

**Published:** 2020-06-23

**Authors:** Alexander Lischke, Rike Pahnke, Anett Mau-Moeller, Robert Jacksteit, Matthias Weippert

**Affiliations:** ^1^Department of Psychology, University of Greifswald, Greifswald, Germany; ^2^Department of Sport Science, University of Rostock, Rostock, Germany; ^3^Department of Orthopaedics, University Medicine Rostock, Rostock, Germany

**Keywords:** heartbeat detection, interoception, reappraisal, suppression, sex differences

## Abstract

Over the last years, there has been a resurge in the interest to study the relationship between interoception and emotion. By now, it is well established that interoception contributes to the *experience* of emotions. However, it may also be possible that interoception contributes to the *regulation* of emotions. To test this possibility, we studied the relationship between interoception and emotion regulation in a sample of healthy individuals (*n* = 84). We used a similar heartbeat detection task and a similar self-report questionnaire for the assessment of interoceptive accuracy and emotion regulation as in previous studies. In contrast to previous studies, we differentiated between male and female individuals in our analyses and controlled our analyses for individual characteristics that may affect the relationship between interoceptive accuracy and emotion regulation. We found sex-differences in interoceptive accuracy and emotion regulation that amounted to a sex-specific relationship between interoceptive accuracy and emotion regulation: Whereas interoceptive accuracy was related to reappraisal but not to suppression in male individuals, interoceptive accuracy was unrelated to reappraisal and suppression in female individuals. These findings indicate that the relationship between interoception and emotion regulation is far more complex than has been suggested by previous findings. However, these findings nonetheless support the view that interoception is essential for both, the *regulation* and *experience* of emotions.

## Introduction

More than two centuries ago, William James challenged contemporary beliefs about emotions by claiming that the perception of autonomic changes is an essential part of an emotional experience (James, 1884). Although James has been heavily criticized for his claims (Cannon, 1927), the idea that emotional experiences involve the perception of autonomic changes persisted over the centuries. Nowadays, it is widely acknowledged that the perception of autonomic changes, in conjunction with a context-dependent interpretation of these changes, forms the basis of emotional experiences (Schachter and Singer, 1962). However, the perception and interpretation of autonomic changes may not only be relevant for the *experience* of emotions but also the *regulation* of emotions (Critchley and Garfinkel, 2017). An accurate perception and interpretation of autonomic changes may lead to emotional experiences that are easy to understand and to regulate, whereas an inaccurate perception and interpretation of autonomic changes may lead to emotional experiences that are difficult to understand and to regulate. Accumulating evidence suggests that this is indeed the case (Critchley and Garfinkel, 2017). Most of the evidence has been gathered in studies that used objective measures of interoceptive accuracy and subjective measures of emotion regulation to investigate the relationship between the perception and interpretation of autonomic changes and the regulation of emotional experiences (Füstös et al., 2013; Weiss et al., 2014; Kever et al., 2015). These studies revealed a positive relationship between interoceptive accuracy and emotion regulation, implying that individuals who were more accurate in interoception were also more efficient in the regulation of their emotional experiences. Interestingly, the positive relationship between interoceptive accuracy and emotion regulation was unaffected by the type of strategy that was employed to regulate the emotional experiences (Füstös et al., 2013; Weiss et al., 2014; Kever et al., 2015). Individuals who were more accurate in interoception were generally more efficient in the regulation of their emotional experiences (Füstös et al., 2013; Kever et al., 2015), regardless whether they re-interpreted the emotional experience *via* reappraisal strategies (Gross and John, 2003) or inhibited the emotional experience *via* suppression strategies (Gross and John, 2003). However, it remained unclear whether this was similarly true for male and female individuals because sex differences were not explored (Füstös et al., 2013; Weiss et al., 2014; Kever et al., 2015). As other studies revealed differences in interoceptive accuracy between male and female individuals (Bornemann and Singer, 2017; Grabauskaitė et al., 2017), it may be possible that interoceptive accuracy was differentially related to emotion regulation in male and female individuals (Füstös et al., 2013; Weiss et al., 2014; Kever et al., 2015).

In the present study, we investigated whether the relationship between interoceptive accuracy and emotion regulation differed between male and female individuals. We assessed individuals’ interoceptive accuracy with the same heartbeat detection task that has been used in previous studies (Füstös et al., 2013; Weiss et al., 2014; Kever et al., 2015). Individuals’ emotion regulation was determined on basis of a widely used self-report questionnaire that assessed similar aspects of emotion regulation as in previous studies (Füstös et al., 2013; Weiss et al., 2014; Kever et al., 2015), namely reappraisal and suppression. As previous studies employed a correlation-based approach to data analysis (Füstös et al., 2013; Weiss et al., 2014; Kever et al., 2015), we also used correlation-based methods to compare the relationship of interoceptive accuracy and emotion regulation between male and female individuals. Individual characteristics that are known to affect the relationship between interoceptive accuracy and emotion regulation were under statistical control during data analysis, which has not been done in previous studies (Füstös et al., 2013; Weiss et al., 2014; Kever et al., 2015). Of particular interest were individual characteristics such as age (i.e., suppression use is more pronounced and interoceptive accuracy is more compromised in older than younger individuals; Khalsa et al., 2009b; Shiota and Levenson, 2009), body mass index (i.e., suppression use is more pronounced and interoceptive accuracy is more compromised in non-lean than lean individuals; Rouse et al., 1988; Andrei et al., 2018), psychopathology (i.e., suppression use is more pronounced and interoceptive accuracy is more compromised in mentally disordered than healthy individuals; Pollatos et al., 2009; Aldao and Nolen-Hoeksema, 2010), autism (i.e., suppression use is more pronounced and interoceptive accuracy is more compromised in autistic than non-autistic individuals; Samson et al., 2012; Garfinkel et al., 2016), empathy (i.e., suppression use is more pronounced and interoceptive accuracy is more compromised in non-empathetic than empathetic individuals; Lebowitz and Dovidio, 2015; Shah et al., 2017) and alexithymia (i.e., suppression use is more pronounced and interoceptive accuracy is more compromised in alexithymic than non-alexithymic individuals; Herbert et al., 2011; Laloyaux et al., 2015). These individual characteristics were assessed with self-report questionnaires. Our study design, thus, allowed us to investigate the relationship between interoceptive accuracy and emotion regulation in male and female individuals with more methodological rigor than in previous studies (Füstös et al., 2013; Weiss et al., 2014; Kever et al., 2015). We expected this relationship to be more pronounced in male than female individuals because interoceptive accuracy has previously been reported to be higher in male than female individuals (Bornemann and Singer, 2017; Grabauskaitė et al., 2017).

## Materials and Methods

### Participants

Eighty-four individuals participated in the study which was part of a larger project investigating the interplay between interoceptive accuracy and emotion processing (Lischke et al., 2020). A screening questionnaire indicated that these individuals were aged between 18–35 years, native speakers and free of mental health problems that required psychotherapeutic treatment at the time of the study. Two individuals did not provide valid data, limiting the number of individuals that could be considered in the statistical analysis to 82 individuals. However, a power analysis (G*Power; Faul et al., 2009) indicated that a sample size of 34 male and 34 female individuals would be large enough to guarantee that meaningful relationships between interoceptive accuracy and emotion regulation could be detected in the statistical analysis (*α* = 0.05, 1-*ß* = 0.80, *r* = 0.40, one-sided correlation analysis). All individuals provided written informed consent to the study procedures that were approved by the local ethics committee and carried out following the Declaration of Helsinki.

### Procedure

Following a debriefing about the study procedure, individuals were seated in a chair and prepared for the heartbeat detection task (Schandry, 1981). After completion of the heartbeat detection task, self-report questionnaires were administered. The questionnaires assessed psychopathology (BSI-18; Franke et al., 2017), alexithymia (Toronto Alexithymia Scale, TAS-20; Franz et al., 2008), autism (Autism Quotient, AQ-10; Allison et al., 2012), empathy (Emotional Contagion Scale, ECS; Doherty, 1997) and emotion regulation in terms of reappraisal and suppression (Affective Style Questionnaire, ASQ; Hofmann and Kashdan, 2010).

### Heartbeat Detection Task

As outlined elsewhere in more detail (Schandry, 1981), individuals were asked to count their heartbeats during three different time intervals (25, 35, 45 s) while their heart rate was recorded with a portable heart rate monitor (Polar Electro Oy, Kempele, Finland). They were not informed about the length of the time intervals and they were not allowed to use any measure that facilitated their task performance. An established algorithm^1^ was used to derive individuals’ interoceptive accuracy from their task performance (Schandry, 1981).

### Statistical Analyses

To account for deviations from normality, non-parametric analyses were performed. Sex-differences in individuals’ demographical (age), anthropometric (body mass index) and psychological (psychopathology, alexithymia, autism, empathy, emotion regulation, and interoception) characteristics were investigated with Mann–Whitney tests (Monte Carlo Simulations with 10,000 samples). Sex-specific correlations between individuals’ interoceptive accuracy and emotion regulation were investigated with Spearman correlations. To obtain unbiased correlation coefficients, partial correlations^2^ were computed that controlled for differences in individuals’ demographical (age), anthropometric (body mass index) and psychological (psychopathology, alexithymia, autism, and empathy) characteristics. The resulting correlation coefficients were compared with one another to confirm possible differences between the respective correlations (Steiger, 1980). The significance level for all analyses was set at *p* ≤ 0.05, two-sided for Mann–Whitney tests and one-sided for Spearman correlations. In addition to the significance values (*p*), effect size measures (*d*, *r, q*) were determined to facilitate the interpretation of the analyses (Cohen, 1992). All analyses were performed with SPSS 24 (SPSS Inc., Chicago, IL, USA).

## Results

### Participant Characteristics

Male and female individuals did not differ in demographical (age: *U* = 736.50, *p* = 0.340, *d* = 0.21; see Table 1) but anthropometric (body mass index: *U* = 258.000, *p* ≤ 0.001, *d* = 1.49; see Table 1) characteristics: Male individuals were as old as female individuals but had a greater body mass index than female individuals. Male and female individuals also differed on certain psychological characteristics: psychopathology (BSI-18-GSI: *U* = 817.50, *p* = 0.833, *d* = 0.05; see Table 1) and autism (AQ-10: *U* = 780.00, *p* = 0.557, *d* = 0.12; see Table 1) was similarly pronounced among male and female individuals but alexithymia was more pronounced among male than female individuals (TAS-20: *U* = 568.00, *p* = 0.012, *d* = 0.58; see Table 1) and empathy was less pronounced among male than female individuals (ECS: *U* = 336.50, *p* < 0.001, *d* = 1.21; see Table 1). Emotion regulation was more pronounced among male than female individuals, with male individuals showing more reappraisal and, at least on a trend level, more suppression than female individuals (ASQ-REA: *U* = 608.00, *p* = 0.029, *d* = 0.49; ASQ-SUP: *U* = 640.50, *p* = 0.058, *d* = 0.42; see Table 1). Interoceptive accuracy was also more pronounced among male than female individuals (IAc: *U* = 593.00, *p* = 0.024, *d* = 0.52; see Table 1).

**Table 1:** Individual characteristics.

	Female individuals (*n* = 41)			Male individuals (*n* = 41)			Test statistic
	*M*	*SD*	Range	*M*	*SD*	Range	*p*
Age (years)	26.00	3.53	20.00–34.00	26.73	4.64	17.00–35.00	0.340
Body mass index (kg/m^2^)	20.85	1.91	18.13–27.24	24.13	2.87	17.59–31.56	0.001***
Autism (AQ-10)	2.39	1.05	0.00–4.00	2.29	1.45	0.00–6.00	0.557
Empathy (ECS)	54.80	5.88	40.00–72.00	49.15	4.79	37.00–59.00	0.001***
Alexithymia (TAS-20)	39.85	10.13	20.00–65.00	44.63	10.38	25.00–67.00	0.012**
Psychopathology (BSI-18-GSI)	0.45	0.40	0.00–1.44	0.38	0.27	0.00–0.94	0.833
Emotion regulation (ASQ)							
Suppression (ASQ-SUP)	2.65	0.71	1.00–4.11	2.92	0.61	1.78–4.00	0.058^†^
Reappraisal (ASQ-REA)	3.43	0.66	2.20–5.00	3.73	0.57	2.60–4.80	0.029*
Interoceptive accuracy (IAc)	0.61	0.18	0.31–0.94	0.70	0.16	0.42–0.98	0.024*

Note. ECS, Emotional Contagion Scale (Doherty, 1997); AQ-10, Autism Quotient 10 (Allison et al., 2012); TAS-20, Toronto Alexithymia Scale 20 (Franz et al., 2008); BSI-18-GSI, Brief Symptom Inventory 18–Global Severity Index (Franke et al., 2017); ASQ, Affective Style Questionnaire (Hofmann and Kashdan, 2010); ASQ-SUP, Affective Style Questionnaire—Suppression (Hofmann and Kashdan, 2010); ASQ-REA, Affective Style Questionnaire—Reappraisal (Hofmann and Kashdan, 2010); IAc, Interoceptive accuracy (Schandry, 1981). ***p ≤ 0.001, **p ≤ 0.01, *p ≤ 0.05, ^†^p ≤ 0.10.

### Relationship Between Interoceptive Accuracy and Emotion Regulation

**Figure 1:**
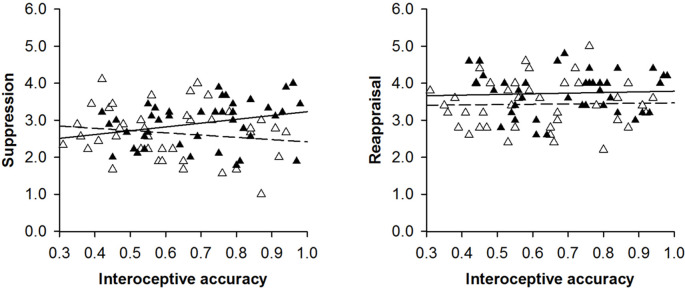
Scatterplots with lines of best fit demonstrating the relationship between interoceptive accuracy and reappraisal or suppression among female (white triangles and dashed lines) and male (black triangles and solid lines) individuals. For visualization purposes, raw data was used.

Among female individuals, interoceptive accuracy was uncorrelated with emotion regulation: interoceptive accuracy neither correlated with reappraisal (ASQ-REA: *r*_(33)_ = 0.04, *p* = 0.400; see Figure 1) nor with suppression (ASQ-SUP: *r*_(33)_ = −0.03, *p* = 0.423; see Figure 1). Among male individuals’, on the contrary, interoceptive accuracy correlated with emotion regulation: interoceptive accuracy correlated with suppression (ASQ-SUP: *r*_(33)_ = 0.35, *p* = 0.02; see Figure 1) but not with reappraisal (ASQ-REA: *r*_(33)_ = −0.19, *p* = 0.141; see Figure 1). A comparison of the correlation coefficients confirmed that interoceptive accuracy correlated with emotion regulation among male but not female individuals (ASQ-SUP: *z* = 1.72, *p* = 0.043, *q* = 0.39; ASQ-REA: *z* = 0.07, *p* = 0.472, *q* = 0.02) and that the correlation between interoception and emotion regulation among male individuals was true for suppression but not for reappraisal (*z* = 1.61, *p* = 0.054, *q* = 0.30).

## Discussion

In the present study, we investigated whether interoceptive accuracy was differentially related to emotion regulation in male and female individuals. Interoceptive accuracy was assessed with a well-established heartbeat detection task and emotion regulation was assessed with a widely used self-report questionnaire that differentiated between reappraisal and suppression. The relationship between interoceptive accuracy and the different emotion regulation strategies was investigated with correlation-based analyses. These analyses revealed sex- and strategy-specific correlations between interoceptive accuracy and emotion regulation. In male individuals, interoceptive accuracy correlated with suppression but not with reappraisal. In female individuals, on the contrary, interoceptive neither correlated with suppression nor with reappraisal. This pattern of correlations emerged in a series of well-powered and hypothesis-driven analyses, which involved a formal comparison of the respective correlation coefficients. The resulting test statistics corresponded to medium effect sizes, implying that we found a robust and meaningful relationship between interoceptive accuracy and emotion regulation in terms of suppression but not reappraisal in male as compared to female individuals. The positive nature of this relationship suggests that male individuals with high interoceptive accuracy were more likely to use suppression for emotion regulation than male individuals with low interoceptive accuracy.

Previous studies also reported a positive relationship between interoceptive accuracy and emotion regulation (Füstös et al., 2013; Weiss et al., 2014; Kever et al., 2015). However, not all of these studies differentiated between reappraisal and suppression in their analyses, which complicates a comparison of the respective findings. The findings of the present study are, nonetheless, broadly consistent with the findings of those studies that performed similar analyses (Füstös et al., 2013; Kever et al., 2015). These studies found a relationship between interoceptive accuracy and suppression that was similar to the one that was found in the present study (Kever et al., 2015). However, these studies also found a relationship between interoceptive accuracy and reappraisal (Füstös et al., 2013; Kever et al., 2015), which was not found in the present study. There are several methodological differences between these studies that may account for the divergence of findings (e.g., differences in the size and composition of the samples, differences in the assessment of interoceptive accuracy and emotion regulation, differences in the analysis of the relationship between interoceptive accuracy and emotion regulation). One of the most striking differences is the differentiation between male and female individuals in the analyses (Füstös et al., 2013; Weiss et al., 2014; Kever et al., 2015), which has only been done in the present study. Sex-differences in interoceptive accuracy and emotion regulation have already been shown in previous studies (Graser et al., 2012; Bornemann and Singer, 2017; Erreygers and Spooren, 2017; Grabauskaitė et al., 2017): Male individuals were more accurate in interoceptive accuracy (Bornemann and Singer, 2017; Grabauskaitė et al., 2017) and more engaged in suppression and reappraisal for emotion regulation (Graser et al., 2012; Erreygers and Spooren, 2017) than female individuals. We found similar sex-differences in interoceptive accuracy and emotion regulation in the present study, indicating the need to consider sex-differences when analyzing the relationship between interoceptive accuracy and emotion regulation. However, male and female individuals may also differ in other characteristics that affect the relationship between interoceptive accuracy and emotion regulation, like, for example, empathy (Doherty, 1997), alexithymia (Franz et al., 2008), autism (Baron-Cohen et al., 2001) or psychopathology (Franke et al., 2017). Consequently, we not only considered individuals’ sex in our analyses but also controlled our analyses for differences in individuals’ sociodemographic (age), anthropometric (body mass index) and psychological (psychopathology, autism, alexithymia, and alexithymia) characteristics. As could be expected on basis of other studies showing more interoceptive accuracy and more suppression in male as compared to female individuals (Abler and Kessler, 2009; Graser et al., 2012; Bornemann and Singer, 2017; Grabauskaitė et al., 2017), we found interoceptive accuracy to be related to suppression but not reappraisal in male as compared to female individuals. Considering the methodological rigor that we applied to these analyses, it seems reasonable to assume that the divergent findings of the present and previous studies are due to methodological differences in data analysis (Füstös et al., 2013; Weiss et al., 2014; Kever et al., 2015). It should be noted, however, that the present and previous studies used correlation-based methods for data analysis. As a consequence, we cannot make causal inferences about the relationship between interoceptive accuracy and suppression or reappraisal in male and female individuals. We, thus, recommend the use of other methods in future studies. Future studies that employ experimental methods, like, for example, the use of pharmacological agents for the manipulation of interoceptive and emotional processes (Khalsa et al., 2009a) or the use of emotion regulation tasks for the manipulation of interoceptive and emotional processes (Füstös et al., 2013), may help to gain more insights into the sex- and the strategy-specific relationship between interoceptive accuracy and emotion regulation.

Notwithstanding these methodological considerations, the findings of the present study can also be explained on basis of theoretical considerations that pertain to the definition of the different emotion regulation strategies (Gross and John, 2003): Reappraisal involves a re-interpretation of emotional experiences that takes place at the cognitive level, whereas suppression involves an inhibition of emotional experiences that takes place on the autonomic and behavioral level. Based on this definition, it could be expected that reappraisal and suppression engage different but overlapping brain regions for the regulation of emotional experiences. Studies investigating the neural correlates of suppression and reappraisal identified a network of brain regions that comprised prefrontal brain regions like the ventral and dorsal prefrontal cortex or the dorsal anterior cingulate cortex (Goldin et al., 2008; Hayes et al., 2010; Giuliani et al., 2011a) and (para-)limbic brain regions like the amygdala or insula (Goldin et al., 2008; Hayes et al., 2010; Giuliani et al., 2011b). Although most of these brain regions were engaged during both emotion regulation strategies (Goldin et al., 2008; Hayes et al., 2010; Giuliani et al., 2011a,b), the insula was more engaged during suppression than reappraisal in these studies (Goldin et al., 2008; Hayes et al., 2010; Giuliani et al., 2011b). However, the insula has also been shown to be engaged during interoception (Critchley et al., 2004; Pollatos et al., 2007a,b; Zaki et al., 2012; Ronchi et al., 2015), in particular in studies that investigated the relationship between interoceptive and emotional experiences (Critchley et al., 2004; Zaki et al., 2012). Due to the aforementioned differences in insula engagement during suppression and reappraisal (Goldin et al., 2008; Hayes et al., 2010; Giuliani et al., 2011b), it could be expected that interoception, which also involved insula engagement (Critchley et al., 2004; Zaki et al., 2012), would be more related to suppression than to reappraisal. Moreover, it could even be expected that this relationship would be more pronounced for male than female individuals because male individuals have been reported to show more insula engagement than female individuals (Lee et al., 2005; Biswal et al., 2010). These expectations were confirmed in the present study where we found interoceptive accuracy to be related to suppression but not to reappraisal in male as compared to female individuals, presumably due to sex- and strategy-specific differences in insula engagement during the integration of interoceptive and emotional experiences. It should be noted, however, that studies investigating sex- and strategy-specific differences in insula engagement during interoception and emotion regulation are scarce. As a consequence, it remains to be determined in future studies whether sex- and strategy-specific differences in insula engagement in fact account for sex- and strategy-specific relationships between interoception and emotion regulation. Considering the complexity of the processes involved in the regulation and experience of interoceptive and emotional phenomena (Pace-Schott et al., 2019), it may be possible that the interplay between interoception and emotion regulation is far more complex than can be assumed on basis of the present study. To address this issue, future studies are warranted that combine subjective measures (e.g., emotion regulation questionnaires) and objective measures (e.g., recordings of neural and autonomic changes during emotion regulation tasks) of interoception and emotion regulation in their investigations.

Given that we found a relationship between interoception and suppression but not reappraisal in male as compared to female individuals, we asked ourselves whether this relationship would be adaptive or maladaptive for these individuals. As previous studies revealed more mental health problems in individuals who used suppression than reappraisal for emotion regulation (Gross, 1998; Moore et al., 2008; Hofmann et al., 2009; Aldao and Nolen-Hoeksema, 2010; Brans et al., 2013), it may be possible that we found a maladaptive rather than adaptive relationship between interoceptive accuracy and emotion regulation. However, the findings of the aforementioned studies have been challenged by the findings of studies that used a more sophisticated methodology to investigate the effects of different emotion regulation strategies on mental health (Bonanno et al., 2004; Troy et al., 2010; Westphal et al., 2010; Meyer et al., 2012; Kalokerinos et al., 2015). These studies suggest that it may depend on the person- and/or context-related factors whether the use of a particular emotion strategy leads to more or less mental health problems (Kashdan and Rottenberg, 2010; Bonanno and Burton, 2013; Sheppes et al., 2015). Consequently, it may be premature to assume that the relationship between interoceptive accuracy and suppression was maladaptive for the individuals of the present study. As none of these individuals reported mental health problems that required psychotherapeutic treatment, it may even be more likely that this relationship was adaptive rather than maladaptive for these individuals. It should be noted, however, that we relied on self-report measures to determine the presence of mental health problems and the utilization of the mental health system. Future studies should use observer-based measures, like, for example, structured interviews and expert ratings (Lischke et al., 2017), to determine mental health problems and mental health system utilization. These types of studies may help to determine whether the sex- and strategy-specific relationship between interoception and emotion regulation is adaptive or maladaptive for individuals.

Coming to an end, we would like to point out that the findings of the present study replicate and extend the findings of previous studies that also investigated the relationship between interoceptive accuracy and emotion regulation (Füstös et al., 2013; Weiss et al., 2014; Kever et al., 2015). In contrast to previous studies (Füstös et al., 2013; Weiss et al., 2014; Kever et al., 2015), we differentiated between male and female individuals in our analyses and controlled our analyses for individual characteristics that may affect this relationship. As could be expected on basis of previous studies reporting differences in interoception and emotion regulation between male and female individuals (Abler and Kessler, 2009; Graser et al., 2012; Bornemann and Singer, 2017; Grabauskaitė et al., 2017), we found interoceptive accuracy to be related to suppression but not reappraisal in male as compared to female individuals. We, thus, believe that future studies investigating the relationship between interoceptive accuracy and emotion regulation may benefit from employing a similar methodological approach as the one that we employed in the present study. These types of studies may help to further refine the findings of previous studies that suggested a less complex relationship between interoceptive accuracy and emotion regulation than the findings of the present study (Füstös et al., 2013; Weiss et al., 2014; Kever et al., 2015). Nonetheless, the extant findings already support historic and contemporary views that the perception and interpretation of autonomic changes are relevant for the *experience* and *regulation* of emotions (James, 1884; Critchley and Garfinkel, 2017).

## Data Availability Statement

The datasets generated for this study are available on reasonable request to the corresponding author.

## Ethics Statement

The study was reviewed and approved by the Ethics committee of the University of Rostock. The participants provided their written informed consent to participate in the study.

## Author Contributions

AL, RP, and MW designed the study. AM-M and RJ collected the data. AL, MW, and RP analyzed the data. AL and RP wrote the manuscript. AM-M, MW, and RJ contributed to writing, reviewing and editing of the manuscript. All authors approved the final version of the manuscript.

## Conflict of Interest

The authors declare that the research was conducted in the absence of any commercial or financial relationships that could be construed as a potential conflict of interest.

## Footnotes

^1^

$$\text{IAc=}\frac{1}{3}\sum \left(1-\frac{\left|n\;{\text{heartbeats}}_{\text{real}}-n\;{\text{heartbeats}}_{\text{counted}}\right|}{n\;{\text{heartbeats}}_{\text{real}}}\right)$$

^2^For the sake of completeness, full correlations were also computed. As can be seen in the Supplementary Material, similar results were obtained when full instead of partial correlations were used in the analyses.
