# Supplementary material for: Sex-Specific Relationships Between Interoceptive Accuracy and Emotion Regulation
Source: Front Behav Neurosci. 2020 Jun 23;14:67. doi: 10.3389/fnbeh.2020.00067 (PMC7324473; doi:10.3389/fnbeh.2020.00067)
Supplement: Supplementary file 1 [file Table_1.pdf]

# Sex-specific relationships between interoceptive accuracy and emotion regulation

Alexander Lischke[1], PhD; Rike Pahnke[2]; Anett Mau-Moeller[2,3], PhD; Robert Jacksteit[3], PhD & Matthias Weippert[2]

[1]Department of Psychology, University of Greifswald, Greifswald, Germany

[2]Department of Sport Science, University of Rostock, Rostock, Germany

[3]Department of Orthopaedics, University Medicine Rostock, Rostock, Germany

**Corresponding authors**:

Alexander Lischke, Department of Psychology, University of Greifswald, Franz-Mehring-Str. 47, D-17487 Greifswald, Germany. Email: alexander.lischke@uni-greifswald.de

Matthias Weippert, Institute of Sport Science, University of Rostock, Ulmenstr. 69, D-18057 Rostock, Germany. Email: matthias.weippert@uni-rostock.de

**Supplemental Material**

*Relationship between interoceptive accuracy and emotion regulation*

Using full instead of partial correlations to analyze sex-specific correlations between individuals' interoceptive accuracy and emotion regulation revealed similar findings: Whereas interoceptive accuracy neither correlated with reappraisal (ASQ-REA: $r(41) = .05$, $p = .387$; see Figure 1) nor with suppression (ASQ-SUP: $r(41) = -.09$, $p = .287$; see Figure 1) in females, interoceptive accuracy correlated with suppression (ASQ-SUP: $r(41) = .30$, $p = .027$; see Figure 1) but not with reappraisal (ASQ-REA: $r(41) = .05$, $p = .382$; see Figure 1) in males. A comparison of the correlation coefficients confirmed that interoceptive accuracy correlated with suppression but not with reappraisal in male as compared to female individuals (ASQ-SUP: $z = 1.77$, $p = .039$, $q = .41$; ASQ-REA: $z = 0.01$, $p = .495$, $q = .00$). The comparison of the correlation coefficients left open whether interoceptive accuracy correlated more with suppression than with reappraisal use in male individuals ($z = 1.24$, $p = .107$, $q = .27$). However, this comparison of correlation coefficients was not central to the hypotheses of the study regarding more pronounced correlations between interoceptive accuracy and emotion regulation in male than female individuals.
